# Surgical management of retroperitoneal schwannoma complicated with severe hydronephrosis: A case report

**DOI:** 10.1097/MD.0000000000012528

**Published:** 2018-09-28

**Authors:** Liandong Zhang, Ming Gao, Tongdian Zhang, Tie Chong, Ziming Wang, Wenpin Liu, Hecheng Li

**Affiliations:** a Department of Urology, The Second Affiliated Hospital, Xi’an Jiaotong University; b Department of Nephrology, Xi’an No. 4 Hospital, Xi’an, Shaanxi, PR China.

**Keywords:** diagnosis, hydronephrosis, prognosis, retroperitoneal schwannoma, treatment

## Abstract

**Rationale:**

Schwannomas are usually benign tumors arising from well-differentiated schwann cells, which rarely occur in the retroperitoneal space. The lack of specific signs and radiologic imaging characteristics makes preoperative diagnosis rather difficult. Most retroperitoneal schwannomas are benign and the primary treatment choice for retroperitoneal schwannomas is surgical excision, however, the involvement of the urinary system is scarcely reported.

**Patient concerns:**

A 34-year-old woman presented with progressive left abdominal pain and rebound abdominal mass at the left lower quadrant for 1 month. Radiological imaging suggested capsulated solid mass with cystic and necrotic areas in the retroperitoneum accompanied by severe left kidney hydronephrosis and preoperative biopsy result was inconclusive.

**Diagnoses:**

We believe this is a rare case of retroperitoneal schwannoma complicated with severe hydronephrosis.

**Interventions:**

After preparation, the patient underwent laparoscopy exploration and converted to open surgical exploration. The patient accepted complete surgical excision of the retroperitoneal tumor and left kidney. Postoperative pathology diagnosis of the mass was proven to be benign retroperitoneal schwannoma.

**Outcomes:**

Postoperative course of the patient was uneventful and the left abdominal pain was greatly improved. After 12-month follow up, no evidence of recurrence or any other complication including renal failure was observed.

**Lessons:**

Preoperative imaging and preoperative ultrasound-guided biopsy are helpful to make accurate diagnosis. The final diagnosis is based on postoperative histological and immunohistochemical findings. The primary treatment option is complete surgical resection of the retroperitoneal schwannoma and the involved upper urinary system when severe hydronephrosis occured. Local recurrence and overall survival are closely correlated with negative resection margins and pathology types.

## 1 Introduction

Retroperitoneal schwannomas are rare tumors originating from Schwann cells of peripheral nerve sheaths, accounting for 0.5% to 5% of all schwannomas.^[1]^ Retroperitoneal schwannomas may occur in all ages, but are mostly seen in patients aged 20 to 60 years old and dominantly in female.^[2]^ The lack of specific signs and symptoms at the early stage makes it hard to diagnose. At the advanced stage, the main presentations of the retroperitoneal schwannomas includes abdominal pain and palpable abdominal mass, other atypical presentations such as secondary hypertension, hematuria, and renal colic were also reported in the literatures.^[3,4]^

Most retroperitoneal schwannomas are benign and malignant ones are rarely seen. If associated with von Recklinghausen's disease, retroperitoneal schwannomas may undergo malignant transformation^[5]^ and exhibit irregular contours as well as distinct invasiveness into the adjacent tissue, accounting for 5% to 18% of all retroperitoneal schwannomas.^[6]^ Although imaging modalities including ultrasound, computed tomography (CT), and magnetic resonance imaging (MRI) may help to make the preoperative diagnosis, the lack of specific imaging signs make the definitive diagnosis of retroperitoneal schwannomas inevitably depend on intraoperative frozen section and postoperative histological examination.

The primary treatment choice for retroperitoneal schwannomas is surgical excision due to insensitivity of these tumors to radiation and chemotherapy^[7]^ and intraoperative frozen section is critical to determine the extent of surgical management, especially for malignant ones. Other surgical approaches including laparoscopic surgery and robotic laparoscopic resection exhibit promising future in the treatment but the choice of surgical management may largely rely on the size and location of tumors.^[2]^

For most cases, the diameter of retroperitoneal schwannomas was <5 cm and the involvement of the urinary system is scarcely reported. Herein we present a case of retroperitoneal schwannomas complicated with severe hydronephrosis and review the literatures, to provide an overall understanding of the diagnosis, treatment, and prognosis of retroperitoneal schwannomas. Written informed consent was obtained from both patients for publication of this case report and accompanying images.

## 2 Case report

A 34-year-old woman was admitted to department of urology in April 2017 with chief complaint of progressive left abdominal pain for 1 month and can be alleviated by oral pain killer, not accompanied by urinary frequency, urgency, nausea, and vomiting. Physical examination revealed rebound, tender, and large abdominal mass was palpable at left upper quadrant.

**Figure 1:**
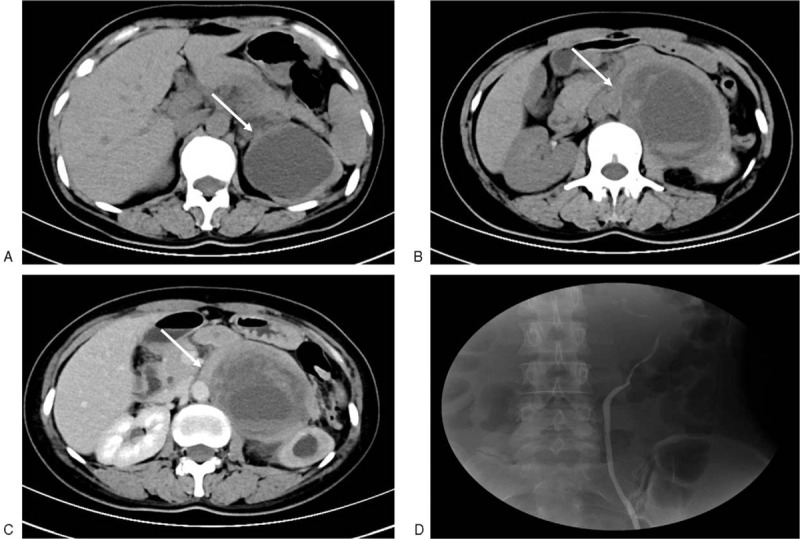
Radiologic features of the retroperitoneal mass. (A) CT scan revealed severe hydronephrosis of the left kidney (arrow head); (B and C) the presence of a 6 × 9 × 8 cm enhancing capsulated solid mass with cystic and necrotic areas in the retroperitoneum(arrow head); (D) left pelvis and upper ureter were not shown in retrograde pyelography.

Laboratory tests showed normal renal function and elevated carbohydrate antigen 72–4 (CA72–4) level. Dynamic renal imaging revealed that glomerular filtration rate (GFR) of left and right kidney was 3.8 and 62.93 mL/min, respectively. Computed tomography urography (CTU) (Philips Brilliance 64 CT scanner, Philips Medical Systems Co., Ltd, USA) was performed and confirmed the presence of a 6 × 9 × 8 cm enhancing capsulated solid mass with cystic and necrotic areas in the retroperitoneum (Fig. 1B,C), compressing left ureter and causing severe hydronephrosis (Fig. 1A). Retrograde pyelography was done, and the left pelvis and upper ureter were not shown, further indicating the ureter blockage due to mass compression.

Transabdominal ultrasound-guided biopsy of the mass was performed preoperatively, and the pathology diagnosis was inconclusive, showing aggregation of fibrillary, elongated cells, and no cellular atypia was visible. Then the patient was scheduled for laparoscopy exploration and open surgical exploration as alternative. Considering the huge volume and abundant blood supply of the mass, adequate volume of blood products was prepared. In case intestine resection might be performed during the operation, bowel preparation and cleansing enema was performed preoperatively.

After preparation, the patient underwent laparoscopy exploration. Intraoperatively, the retroperitoneal mass was found closely adhered to adjacent tissue, the limited space made it hard to operate and converted to open surgical exploration. The encapsulated mass, measuring 6 × 9 × 8 cm, was densely adherent to mesentery and left kidney. The left ureter was located left rear to the mass and the small intestine was squeezed to the periphery of the mass. Following the separation of the mass from adjacent tissues, the mass was completely excised from retroperitoneum. Then the artery, vein of left kidney and the left ureter were separated and ligated separately. The blood loss during the excision was estimated to be around 400 mL.

**Figure 2:**
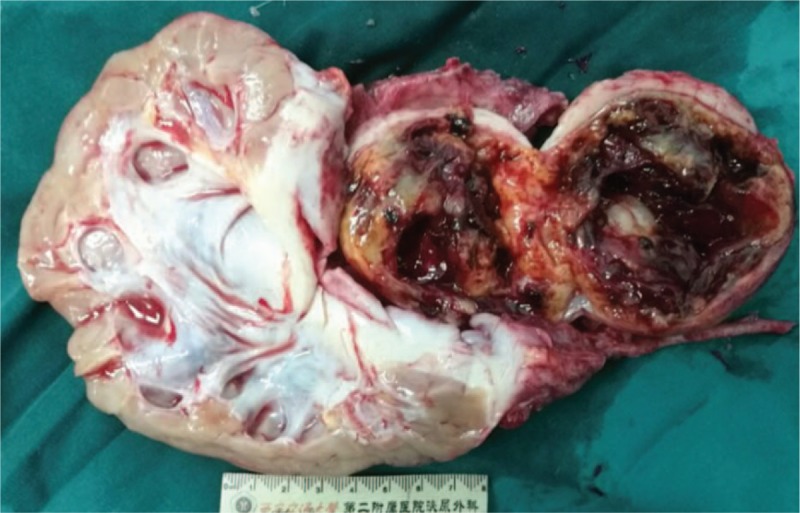
Macroscopical specimens of left kidney and retroperitoneal schwannoma. The mass was round and well circumscribed with areas of necrosis and hemorrhage in the center. The left kidney showed thinning of the renal parenchyma and dilation of the renal pelvis and calyces.

Macroscopically, the well-circumscribed mass was round and yellowish with areas of necrosis and hemorrhage in the center, outer surface was smooth and no sign of invasion was observed. The left kidney showed thinning of the renal parenchyma and dilation of the renal pelvis and calyces (Fig. 2). The specimens were then fixed in 10% buffered-formalin, embedded in paraffin, and cut into 5 mm.

**Figure 3:**
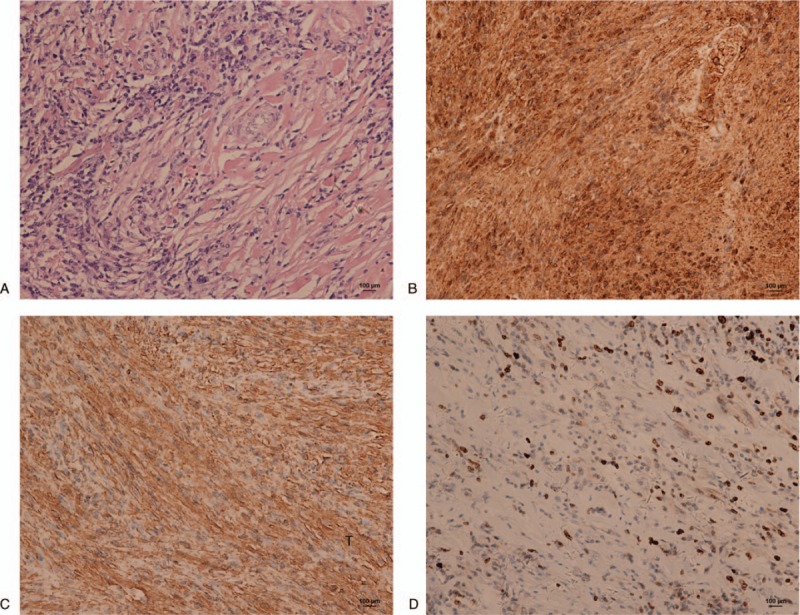
Hematoxylin and eosin staining and immunohistochemical staining of retroperitoneal schwannoma. (A) HE staining revealed pindle-shaped cells in a typical palisading pattern and areas of myxoid and degenerative tissue with fewer cells, no atypical large nuclei and mitosis were observed; (B–D) immumohistochemical staining showed positivity for S-100 (B), vimentin (C), and Ki67 (D). 200 × bar = 100 μm.

Histopathological examination of specimens revealed areas of spindle-shaped cells in a typical palisading pattern and areas of myxoid and degenerative tissue with fewer cells (Fig. 3A). No atypical large nuclei or mitosis was observed. Immunohistochemical staining showed cellular positivity for S-100, vimentin and Ki67 (sporadic +) (Fig. 3B–D). The final diagnosis was benign retroperitoneal schwannoma.

Postoperative course of the patient was uneventful and the left abdominal pain was greatly improved. At the 12-month follow-up, no evidence of recurrence or operation-correlated complication was observed.

## 3 Discussion

Schwannomas are nerve sheath tumors mostly affecting head, neck, and the extremities, among which retroperitoneal schwannomas comprise 0.5% to 12% of all retroperitoneal tumors.^[8]^ Retroperitoneal schwannoma was first reported by Stallworthy in 1944^[9]^ and most cases were reported in population aged 20 to 60 years, in which female had higher morbidity rate than male.

The high flexibility of retroperitoneal space makes retroperitoneal schwannoma lack specific symptoms at early stage and presentation may vary from abdominal mass, flank pain to incidental findings at advanced stage, resulting in the delayed diagnosis and treatment.^[6]^ The 34-year-old female patient in this study also presented atypical symptoms and giant retroperitoneal mass complicated with severe hydronephrosis. Involvement of the urinary tract was rarely reported. Ozbir et al^[10]^ reported one case of giant retroperitoneal schwannoma complicated with bilateral hydronephrosis, no recurrence was found, and bilateral hydronephrosis was decreased after surgical excision of the tumor.

On gross appearance, schwannomas are usually solitary, well circumscribed, firm, and smooth-surfaced tumors,^[11]^ which frequently undergo secondary changes including hemorrhage, cysts, and calcification.^[12]^ Histologically, retroperitoneal schwannoma is characterized by alternating Antoni A and Antoni B areas. Clusters of compact elongated bipolar spindle cells arranged in a palisading pattern are contained in Antoni A areas, whereas Antoni B areas are characteristic by loosely arranged cells in a myxoid background.^[13]^ However, in large retroperitoneal and pelvic schwannomas, uniform spindle cell appearance without independent Antoni A and Antoni B areas was reported.^[14]^ Immunohistologically, strong and diffuse staining of S-100 protein in schwannomas cells cytoplasm is essential for the exact diagnosis of retroperitoneal schwannoma.^[15]^ The diagnosis of malignant retroperitoneal schwannoma may be suggested if mitotic figures, nuclear atypia and blood vessel infiltration are observed histologically.^[16]^

Preoperative radiologic examination plays a vital role in the diagnostic evaluation. As revealed by the computed tomography scan, margin of the retroperitoneal schwannoma is usually well defined, smooth and sharp. Low or mixed attenuation with cystic necrotic central area can also be observed, which occur more commonly in retroperitoneal schwannomas compared with the other retroperitoneal tumors.^[17]^ In this case, degenerative changes including hemorrhage, and hyalinization were also observed. Magnetic resonance imaging provides more accurate preoperative diagnosis compared to CT and ultrasound. The main MRI manifestation of retroperitoneal schwannoma includes hypointensity intensity on T1-weighted images and hyperintensity on T2-weighted images, which may also be affected by the microscopic arrangement of Antoni A and Antoni B areas.^[18]^ The malignant cases usually exhibit mixed intensity on both T1- and T2-weighted images. Although these findings are characteristic, they are not specific to make the preoperative diagnosis of retroperitoneal schwannoma.

The differential diagnoses with schwannomas include fibrosarcoma, liposarcoma, ganglioneuroma, which have similar findings on CT and MRI scan.^[19]^ Preoperative misdiagnosis of retroperitoneal schwannomas is not always uncommon, emphasizing the necessarity of ultrasound guided biopsy preoperatively. However, cellular pleomorphism in areas of degeneration may lead to inaccurate diagnosis but it is still helpful in suspected malignant lesions.^[20]^ In this case, the transabdominal ultrasound-guided biopsy of the mass showed aggregation of fibrillary, elongated cells and no cellular atypia was visible, consisting with the final pathology examination. Considering malignancy cannot be excluded preoperatively and even intraoperative frozen section cannot provide accurate pathology diagnosis, it is recommended that the management of retroperitoneal schwannomas is complete surgical excision with negative soft tissue margins.^[21]^ However, controversies still exist about whether excision of adjacent tissue and viscera is necessary or not. Some authors suggested that simple enucleation or partial excision without removal of adjacent organs was sufficient because of the benign nature for most cases and loss of adjacent organs may influence the prognosis.^[6,11]^ In this case, preoperative GFR of left was 3.8 mL/min and the left pelvis and upper ureter were not shown by retrograde pyelography, suggesting the possibility of tumor invasiveness. During operation, the mass was found to be tightly adherent to the left ureter, and we performed the resection of left kidney, left ureter, and the mass.

Prognosis of benign retroperitoneal schwannomas is extremely good and recurrence is rarely reported, but close follow-up is necessary after removal of benign retroperitoneal schwannomas. Surgical resection is recommended if recurrence occurred. Due to the lack of sensitivity to radiation and chemotherapy, adjuvant therapy is not recommended.^[21]^ In recent years, laparoscopic excision^[22]^ and robotic resection^[23]^ emerge as promising surgical approaches, but the location and the size of tumors may affect the choice of surgical approaches. In this case, we performed laparoscopy exploration initially, but finally converted to open surgical exploration as a result of the limited space and close relationship with surrounding tissue. After 12-month follow-up, no evidence of recurrence or any other complication including renal failure was observed; further follow-up is still required in case of postoperative recurrence.

## 4 Conclusion

We reported a rare case of retroperitoneal benign schwannoma. Preoperative imaging and preoperative ultrasound-guided biopsy are helpful to make accurate diagnosis. The final diagnosis is based on postoperative histological and immunohistochemical finding. The primary treatment option is complete surgical resection; intraoperative frozen sections may help to define the margin of excision. Local recurrence and overall survival are closely correlated with negative resection margins and pathology types.

## Acknowledgments

The authors thank Dr. Peng Zhang (Department of Urology, The Second Affiliated Hospital, Xi’an Jiaotong University) for assistance and various suggestions with respect to the case analysis, and also thank Dr. Jun Yang (Department of Pathology, The Second Affiliated Hospital, Xi’an Jiaotong University) for his kind technical support.

## Author contributions

**Investigation:** Ming Gao, Wenpin Liu.

**Project administration:** Tongdian Zhang.

**Supervision:** Tie Chong.

**Writing – original draft:** Liandong Zhang.

**Writing – review and editing:** Ziming Wang, Hecheng Li.
